# Trans-arterial chemoembolization and external beam radiation therapy for treatment of hepatocellular carcinoma with a tumor thrombus in the inferior vena cava and right atrium

**DOI:** 10.1186/s40644-015-0043-3

**Published:** 2015-05-26

**Authors:** Feng Duan, Wei Yu, Yan Wang, Feng-yong Liu, Peng Song, Zhi-jun Wang, Jie-yu Yan, Kai Yuan, Mao-qiang Wang

**Affiliations:** Department of Interventional Radiology, the General Hospital of Chinese People’s Liberation Army, Beijing, 100853 China; Department of Radiotherapy, the General Hospital of Chinese People’s Liberation Army, Beijing, 100853 China

**Keywords:** Chemoembolization, Radiotherapy, Hepatocellular carcinoma, Inferior vena cava, Right atrium, Thrombus

## Abstract

**Background:**

Hepatocellular carcinoma (HCC) with a tumor thrombus in the inferior vena cava (IVC) and right atrium (RA) rarely occurs and is usually associated with extremely poor prognosis, we carried out this study to evaluate the efficacy and safety of a combination of trans-arterial chemoembolization (TACE) and external beam radiation therapy (EBRT) in the treatment of HCC with a tumor thrombus in the IVC and RA.

**Methods:**

From September 2005 to September 2008, 11 cases of HCC with a tumor thrombus in the IVC and RA were treated with a combination of TACE and EBRT. Clinical adverse events, laboratory toxicity, and survival were retrospectively studied.

**Results:**

Thirty-one interventional procedures were conducted and EBRT was performed 11 times. All treatments were successful and without significant complications. No severe adverse effects were observed. The median survival time of the 11 cases was 21.0 months. One patient was monitored for 97 months and no recurrence was observed.

**Conclusion:**

The combination of TACE and EBRT can be safely performed and may improve the prognosis of the HCC cases with a tumor thrombus in the IVC and RA.

## Background

Hepatocellular carcinoma (HCC) is the fifth most commonly diagnosed cancer and the third most common cause of cancer-related death worldwide [1, 2]. Although vascular invasion is a feature of HCC, most of the cases are HCC with portal vein tumor thrombus [3, 4]. HCC with an extension to the inferior vena cava (IVC) and right atrium (RA) rarely occurs and is usually associated with extremely poor prognosis.

HCC that has invaded into the IVC and RA is usually treated with surgery, radiotherapy (RT), trans-arterial chemoembolization (TACE), and chemotherapy. However, the standard treatment modality has not been established. Although TACE is a relatively safe treatment procedure, its therapeutic effect, especially for tumor thrombi in IVCs and RAs, remains unsatisfactory [5, 6].

A few studies have shown that the combination of TACE with RT may improve therapeutic responses [7, 8]. Therefore, in the present study, we retrospectively investigated 11 HCC patients with an IVC and RA tumor thrombus who underwent the combination treatment of trans-arterial chemoembolization (TACE) and external beam radiation therapy (EBRT). In our combination therapy protocol, TACE was used to treat intrahepatic lesions and tumor thrombus, and EBRT was exclusively applied to the tumor thrombus in the IVC and RA. The purpose of the present study was to demonstrate the safety and efficacy of the combination of TACE and EBRT in the treatment of patients with highly advanced HCC with a metastatic IVC and RA tumor thrombus.

## Methods

### Patients and treatment protocol

Between September 2005 and September 2008, a total of 11 patients who were diagnosed with advanced HCC with IVC and RA tumor thrombus were treated by a combination of TACE and EBRT. The retrospective analysis of the data was approved by Chinese PLA General Hospital investigational review board.

Pre-treatment evaluation consisted of a complete history and physical examination, blood cell count, liver and renal panels, AFP value, computed tomography (CT) scan of the chest, tri-phase CT scan or dynamic magnetic resonance imaging (MRI) of the liver, and total body bone scan. Fluorodeoxyglucose positron emission tomography (PET) or PET/CT scans were optional.

Treatments were performed when no contraindications were identified. The treatment protocol started with 2–3 sessions of TACE (interval: 1 month), depending on the intrahepatic tumor burden. Two weeks after TACE, EBRT was conducted to target the IVC and RA tumor thrombus. Patients were followed up every 3 months to check whether new tumor lesions in the liver had developed. When lesions were detected, TACE was performed again to control intrahepatic lesions.

### Treatment

All TACE procedures were performed using digital subtraction angiography (DSA) guidance. After a routine preoperative preparation, TACE was performed under sterile conditions, with the patient under local anesthesia. The right femoral artery was cannulated using a 4 F vascular sheath (Radifocus Introducer II; Terumo Corp., Japan) by Seldinger’s technique. Selective angiography of the celiac artery, superior mesenteric artery and inferior phrenic artery was performed using a 4 F hepatic artery catheter (HE, Terumo Corp., Japan) inserted through the vascular sheath. Maximum catheter selectivity of the hepatic artery and inferior phrenic artery was achieved using a microcatheter (Progreat, Terumo Corp., Japan), with administration from the afferent branch to the tumor lesion. Drug dosages per procedure varied, ranging from 6–20 mL for lipiodol (Guerbet Corp., France), 30–50 mg of doxorubicin (Pfizer Pharmaceuticals Ltd, USA), 100–150 mg oxilaplatin (Sanofi Pharmaceuticals Co., Ltd, France), and 8–12 mg mitomycin (Zhejiang Hisun Pharmaceutical Co. Ltd, China), depending on the size of the tumor lesion and laboratory results. Lipiodol-chemotherapeutic agents were administered until stasis, minimizing reflux into non-target vessels. Injection was continued until near stasis was observed in the artery directly feeding the tumor (i.e., the contrast column should clear within 2–5 heartbeats). Gelatin sponge or polyvinyl alcohol particles (PVA, 500–700 μm, COOK Corp., USA) was injected as supplement when necessary.

Three days after the procedure, patients were administered polyenephosphatidylcholine (465 mg, iv, qd, Chengdu Tiantaishan Pharmaceutical Co., China) and glutathione (1,800 mg, iv, qd, LaboratorioFarmaceutico C.T.S.R.L, Italy) to normalize liver function, as well as pain killers and an anti-emetic. On the 3rd day after the completion of TACE, the patients were assessed for adverse effects by undergoing a physical examination and laboratory testing consisting of blood cell count, as well as liver and renal panels. The patients were discharged from the hospital when the laboratory results were determined to be within normal range.

EBRT was initiated after the intrahepatic lesions were controlled by TACE. EBRT was initiated 2 weeks after the last session of TACE. All patients underwent three-dimensional conformal radiotherapy (3D-CRT). For radiotherapy planning, the patients underwent contrast-enhanced CT scans in a supine position, with both arms raised above the head. Simulation CT data was transferred to a radiation treatment planning system (Pinnacle, The Philips Medical System, Netherland). The clinical target volume (CTV) included IVC and RA tumor thrombus without primary intrahepatic disease and was defined as the radiographically abnormal areas and noted on the planning CT images from the diagnostic enhanced CT and/or MRI. The 4D-CT and respiratory gating techniques were not used in the present study. Considering setup error and target motion, PTV was determined as the CTV plus 5 mm for the anterior, posterior, medial, and lateral margins, and 10 mm for the superior and inferior margins. Organs at risk (OARs) included the liver, heart, duodenum, spinal cord, and kidneys. Dose constraints for OARs were defined as follows: the mean dose and V35 (Vn, the percentage of volume receiving more than n Gy) of the liver were maintained at < 30 Gy and 50 %, respectively. The mean dose and V50 of the heart were maintained at < 40 Gy and 50 %, respectively. The V50 and V45 of the duodenum were maintained at < 2 % and 25 %, respectively. The maximum dose was maintained < 45 Gy for the spinal cord. The mean dose for each kidney was maintained < 23 Gy. 3D-CRT was delivered by using a linear accelerator with 10-MV X-rays, 3–5 beams. A daily fraction of 2 Gy was administered at five fractions per week to deliver a total dose of 60 Gy.

### Evaluation

Acute and late toxicities from treatments were graded according to NCI-CTCAE version 4.0 [9]. Responses were defined using the mRECIST criteria [10] based on an enhanced CT of the thorax and MRI of the liver.

### Data analyses

SPSS for Windows, version 16.0; SPSS Inc., Chicago, Ill, USA) was used for data analysis. The duration of OS was calculated from the diagnosis of HCC until death or until the date of the last follow-up visit for patients still alive.

## Results

The characteristics of the patients included in the study are presented in Table 1. Radiologically confirmed complete response (CR) of the tumor thrombus in IVC and RA was achieved in all cases. Radiologically confirmed CR, partial response (PR), stable disease (SD), and disease progression (PD) for intrahepatic disease were observed in 2, 6, 2, and 1 patient, respectively, after 2–5 sessions of TACE. Metastases to organs other than IVC/RA during treatment were observed in 9 patients: 6 had lung metastasis (1–6 months from the first treatment), 1 had lung (3 months from first treatment) and adrenal metastases (7 months from first treatment), and 2 had bone metastasis (5 and 9 months from first treatment).

**Table 1:** Patient characteristics (at diagnosis of tumor thrombus in IVC/RA

Case	Age	Gender	Etiology	Pathology	Intrahepatic tumor size (cm)	AFP (μg/L)	Survival time (months)	Status
1	54	M	Hepatitis B	Moderately differentiated HCC	7	16.74	21	Dead
2	44	M	Hepatitis B	NA	5	322.11	97	Alive
3	30	F	Hepatitis B	NA	11	1210	11	Lost
4	55	M	Hepatitis B	Poorly differentiated HCC	6	2.01	24	Dead
5	53	M	Hepatitis B	NA	5	1,105	66	Alive
6	40	M	Hepatitis B	NA	6.5	390.68	25	Dead
7	48	M	Hepatitis B	Moderately differentiated HCC	10.5	>24,200	8	Dead
8	61	M	Hepatitis B	NA	10	1,503	9	Lost
9	74	M	Hepatitis B	NA	9.5	903	10	Dead
10	64	M	Hepatitis B	NA	9	1,842	7	Lost
11	48	M	Hepatitis B	NA	6.5	>20,000	36	Dead

Among the 9 patients (81.8 %) with metastases, 7 patients with lung metastasis were treated with systemic chemotherapy (2 patients) and oral administration of sorafenib (5 patients), depending on the financial capacity of the patient. The chemotherapeutic protocol was FOLFOX4: oxaliplatin, 85 mg/m^2^, intravenous (iv.) at day 1; leucovorin (LV), 200 mg/m^2^, iv. at days 1 and 2; 5-fluorouracil (5-FU), 400 mg/m^2^, iv. bolus and then 600 mg/m^2^, iv. for at least 22 h on days 1 and 2. The protocol was repeated every 2 weeks. Follow-up indicated that the treatments resulted in poor response and lung metastases in all 7 patients. One patient with adrenal metastasis was treated by adrenal artery chemoembolization; follow-up images showed that PR was achieved. Two patients with bone metastasis were received symptomatic treatment by bisphosphonate zoledronic acid (4 mg, iv. repeated every month).

**Fig. 1:**
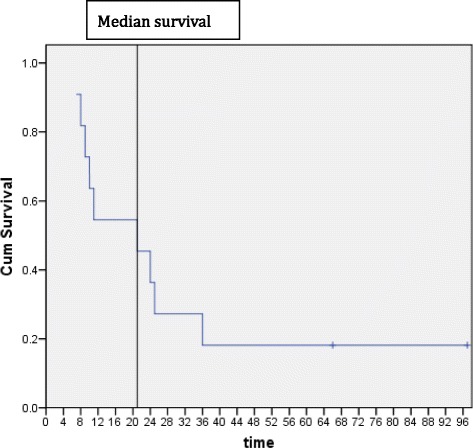
Overall survival of the entire group of patients. Median survival: 21.0 months

The 1- and 3-year overall survival rates of the 11 patients were 54.5 % and 27.3 %, respectively, and the overall median survival duration was 21.0 months (Fig. 1). At the time of this analysis, 6 patients (54.5 %) had died and 3 (27.3 %) patients were lost to follow-up. All patients died of disease progression. The longest survival time was 97 months and remains alive.

Most patients experienced TACE-induced adverse effects, which included pain, nausea, and transient reduction of blood counts. However, severe (grades 3 or 4) adverse effects were not observed.

## Discussion

With the development of treatment for HCC, survival rates significantly improved. However, HCC with metastatic IVC and RA tumor thrombus still has an extremely poor prognosis [11, 12], and no effective therapy has been reported to date. The median survival of patients with metastatic IVC and RA tumor thrombus without effective therapy is 2–3 months [13].

TACE is one of the most commonly used local treatment modalities for unresectable HCC. However, it has relative poor efficacy in resolving tumor thrombi. Numerous clinical studies have demonstrated that the combination of TACE and EBRT may improve therapeutic responses of HCC with PVTT [14, 15]. Therefore, it was reasonable to postulate that a combination of TACE and EBRT may improve prognoses of HCC with a tumor thrombus in the IVC and RA. In the present study, median survival was 21 months, demonstrating that the combination treatment is effective.

**Fig. 2:**
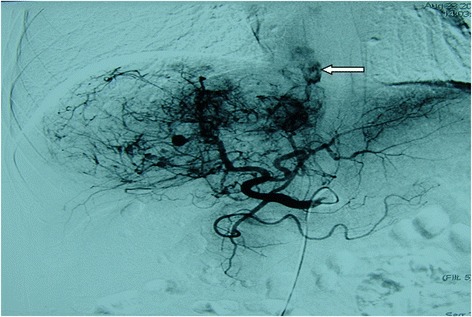
Hepatic artery angiography of a 54-year-old showing a branch of the hepatic artery feeding the IVC/RA tumor thrombus (←)

**Fig. 3:**
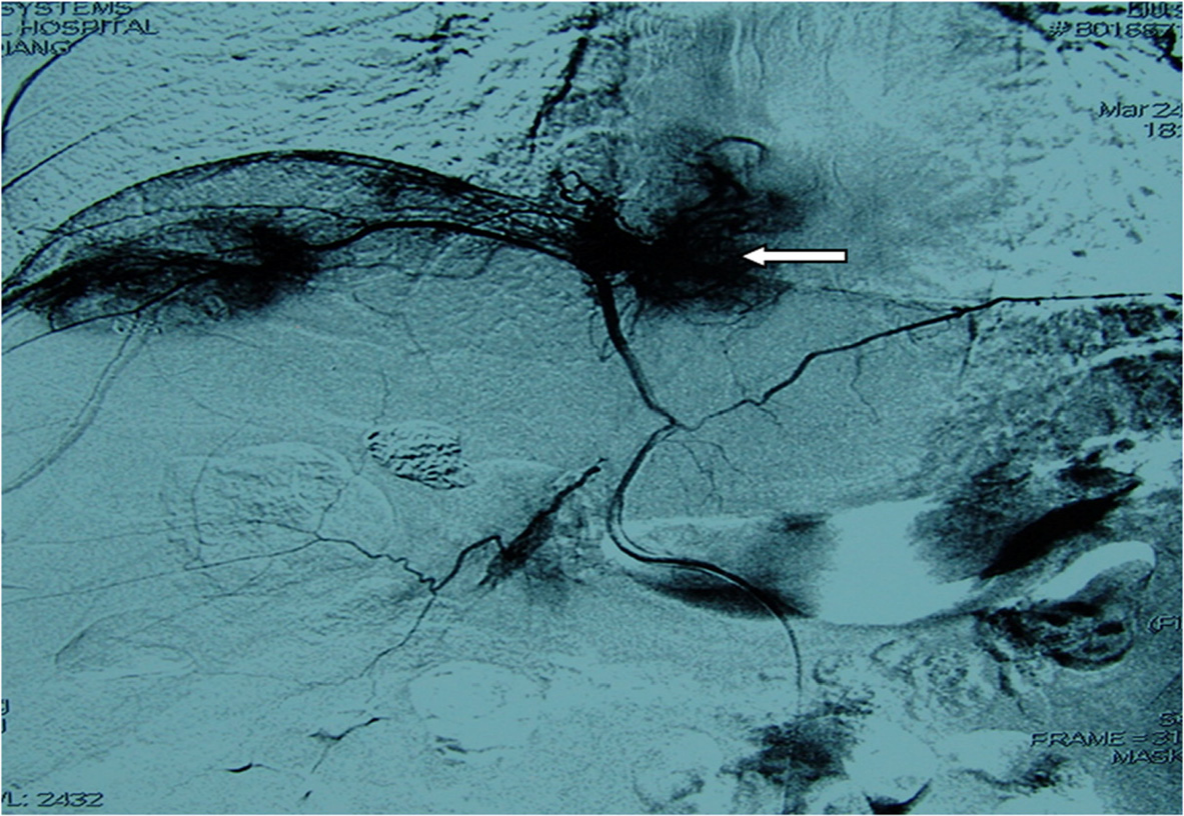
Right inferior phrenic artery angiography of a 55-year-old male showing an IPA-fed IVC/RA tumor thrombus (←)

**Fig. 4:**
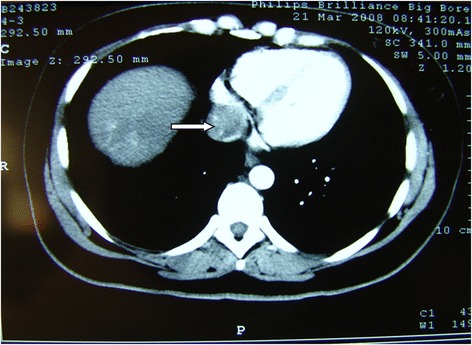
Enhanced CT arterial phase of a 30-year-old female showed filling-defect in IVC/RA (→)

**Fig. 5:**
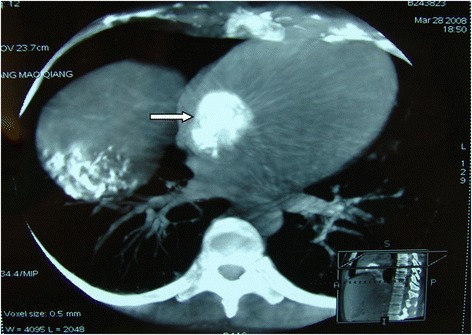
The same patient presented in Fig. 4; C-armed CT showing nice lipiodol deposition in the tumor IVC/RA thrombus after chemoembolization (→)

In the present study, a tumor thrombus in IVC and RA was distinct from a portal vein tumor thrombus in that it has a defined arterial blood supply, which included the hepatic artery (5/11) and inferior phrenic artery (IPA) (6/11). During the TACE procedure, we delivered lipiodol-chemotherapeutic agents to the tumor thrombus via a microcatheter, and follow-up images showed lipiodol deposition (Figs. 2, 3, 4 and 5).

Historically, conventional EBRT has been infrequently used in the treatment of HCC because of low tolerance of the liver for RT. However, recent advances in radiation techniques such as 3D-CRT, image-guided radiotherapy (IGRT), and intensity-modulated radiotherapy (IMRT) have allowed us to deliver high doses of EBRT to the tumor without causing dose-limiting toxicities in the surrounding normal tissue [16, 17]. In the present study, high-dose conformal radiotherapy showed a significant response and acceptable toxicities by excluding the non-tumorous volume of the liver from the target volume.

Poor prognosis of HCC with a tumor thrombus in the IVC/RA is strongly related to a high incidence of metastasis and recurrence [18, 19]. In the present study, most patients developed other organ metastases during treatment, and therapies for metastases including systemic chemotherapy and target therapy showed relatively poor responses. To date, no clear modality in preventing HCC recurrence and metastasis has been established. Therefore, investigations on novel systemic therapies to treat metastases are warranted [20, 21].

It is generally believed that the mechanism of IVC/RA metastases is direct and serves as contiguous extensions of the intrahepatic HCC [17, 22]. The growth of the tumor thrombus involves tightly adhering to the hepatic vein and inferior vena cava. There have been no reports on the dislodgement of the thrombus, and consistent with this, no thrombus dislodgment was noted in our study. However, there is still a risk for thrombus dislodgment; therefore, ultrasound was performed every 3 months to evaluate the stability of thrombus.

The limitations of this study include the relatively small number of patients and the use of a few comparisons that prevented a meaningful analysis of the efficacy of the combination therapy. Because of the low occurrence of the HCC patients with IVC/RA metastases, it is difficult to recruit more patients to perform a perspective comparative study.

## Conclusions

The combination of TACE and EBRT can be safely performed in HCC patients with an IVC or RA tumor thrombus, resulting in a median survival of 21 months. However, metastasis remains an unresolved issue that restricts prognosis. Further investigations on systemic therapies for metastatic tumors should be pursued.

## Consent

Written informed consent was obtained from the patient for the publication of this report and any accompanying images.
